# Supplementary material for: PLOS Pathogens 2016 Reviewer and Editorial Board Thank You
Source: PLoS Pathog. 2017 Mar 20;13(3):e1006278. doi: 10.1371/journal.ppat.1006278 (PMC5358882; doi:10.1371/journal.ppat.1006278)
Supplement: S1 Editor List — (PDF) [file ppat.1006278.s001.pdf]

PLOS | PATHOGENS

PLOS Pathogens would like to thank all those who served on the Editorial Board in 2016:

Umberto Agrimi
Christopher Aiken
Raul Andino
Alex Andrianopoulos
François Balloux
Ralph S. Baric
Jason C. Bartz
Christopher F. Basler
Andreas J. Baumler
Marcel A. Behr
Robert Belshaw
Chris A. Benedict
Nora J. Besansky
Debra E. Bessen
Stephen M. Beverley
Oliver Billker
Paul Birch
Michael J. Blackman
Steven R. Blanke
James B. Bliska
Helena Ingrid Boshoff
Kenneth A. Bradley
William J. Britt
Vern B. Carruthers
Sara Cherry
Ambrose Cheung
Chetan E. Chitnis
Jenifer Coburn
James J. Collins III
Richard C. Condit
Brian K. Coombes
Isabelle Coppens
Laurent Coscoy
Leah E. Cowen
Carolyn B. Coyne
Robert A. Cramer
Bryan R. Cullen
Blossom Damania
Jeffery L. Dangl
Christoph Dehio
Kirk W. Deitsch
Eric Y. Denkers
Isabelle Derré
Ronald C. Desrosiers
Michael S. Diamond
Savithramma P. Dinesh-Kumar
Shou-Wei Ding
Dirk P. Dittmer
Tamara L. Doering
Daniel C. Douek
Simon L. Dove
Rebecca Ellis Dutch
Sabine Ehrt
Nels C. Elde
Michael Emerman
Christian R. Engwerda
David T. Evans
Matthew J. Evans
Marta Feldmesser
Pinghui Feng
Neil M. Ferguson
Ana Fernandez-Sesma
Scott G. Filler
Erik K. Flemington
JoAnne L. Flynn
Sarah M. Fortune
Ron A.M. Fouchier
Daved H. Fremont
Klaus Früh
Michaela U. Gack
Sarah L. Gaffen
Michael Gale Jr.
Denise A. Galloway
Andrea Gamarnik
Shou-Jiang Gao
Adolfo Garcia-Sastre
William C. Gause
Ricardo T. Gazzinelli
William E. Goldman
Marc-Jan Gubbels
Hui-Shan Guo
Kasturi Haldar
Alan R. Hauser
Thomas R. Hawn
Sheng Yang He
Patrick Hearing
Mark T. Heise
Joseph Heitman
Kent L. Hill
Tom C. Hobman
Deborah A. Hogan
Thomas J. Hope
David Horn
Lindsey Hutt-Fletcher
Ralph R. Isberg
Patricia J. Johnson

Robert F. Kalejta
Yoshihiro Kawaoka
James W. Kazura
Kami Kim
Bruce S. Klein
Kimberly A. Kline
Laura J. Knoll
Theresa M. Koehler
Richard A. Koup
Hans-Georg Kräusslich
Damian J. Krysan
Tomoko Kubori
Jens H. Kuhn
Richard J. Kuhn
Paul Francis Lambert
Jean Langhorne
Adam S. Lauring
Benhur Lee
Vincent T. Lee
John M. Leong
Bruce R. Levin
David M. Lewinsohn
Paul M. Lieberman
Jeffrey Lifson
Xiaorong Lin
Paul D. Ling
Manuel Llinás
James B. Lok
P'ng Loke
Richard Longnecker
Guangxiang George Luo
Zhao-Qing Luo
Wenbo Ma
Neil A. Mabbott
David Mackey
Michael H. Malim
Robin Charles May
Alison Anne McBride
Bruce A. McDonald
Grant McFadden
Elizabeth Ann McGraw
Craig Meyers
Virginia L. Miller
Aaron P. Mitchell
Timothy J. Mitchell
Edward Mitre
Edward Mocarski
Denise M. Monack
Ashlee V. Moses
Karen L. Mossman
Maria M. Mota
Ingrid Müller
Karl Münger
Eain Anthony Murphy
Peter D. Nagy
Xavier Nassif
Jay A. Nelson
Janko Nikolich-Žugich
Thomas B. Nutman
Carlos Javier Orihuela
Mary O'Riordan
Jing-hsiung James Ou
Peter Palese
Colin Parrish
Matthew R. Parsek
Edward J. Pearce
Andrew Pekosz
Daniel R. Perez
Andreas Peschel
William A. Petri Jr.
Margaret A. Phillips
Dana J. Philpott
Ted C. Pierson
Alice Prince
Nancy Raab-Traub
Vincent Racaniello
Glenn F. Rall
Glenn Randall
Jason L. Rasgon
Laurie Read
Félix A. Rey
Charles M. Rice
Eleanor M. Riley
Christophe Ritzenthaler
Erle S. Robertson
Susan R. Ross
June L. Round
Craig R. Roy
David Sacks
Nina R. Salama
Padmini Salgame
R. Jude Samulski
Andrea J. Sant
Christopher M. Sassetti
Karla J.F. Satchell
Connie S. Schmaljohn
David S. Schneider
Matthias Johannes Schnell
H. Steven Seifert
Donald C. Sheppard
Barbara Sherry
Aleem Siddiqui
Luis J. Sigal
Anita Sil
Guido Silvestri
Eric P. Skaar
Joe Smith
Dominique Soldati-Favre
Samuel H. Speck
Katherine R. Spindler

Mary M. Stevenson
Xin-zhuan Su
Kanta Subbarao
Bill Sugden
Paul M. Sullam
Surachai Supattapone
Sankar Swaminathan
Ronald Swanstrom
Timothy L. Tellinghuisen
Volker Thiel
Paul G. Thomas
Bart Thomma
Greg Tiao
Guy Tran Van Nhieu
Alexandra Trkola
Heather L. True-Krob
Renée M. Tsolis
Brett Tyler
Raphael H. Valdivia
Kenneth D. Vernick
Marco Vignuzzi
Christopher M. Walker
Aiming Wang
David Wang
David Weiss
Michael R. Wessels
David Westaway
Sean P.J. Whelan
E. John Wherry
David L. Williams
Thomas A. Wynn
Jin-Rong Xu
Dario S. Zamboni
Gongyi Zhang
Jian-Min Zhou
Z. Hong Zhou
Cyril Zipfel
