# Supplementary material for: PLOS Pathogens 2016 Reviewer and Editorial Board Thank You
Source: PLoS Pathog. 2017 Mar 20;13(3):e1006278. doi: 10.1371/journal.ppat.1006278 (PMC5358882; doi:10.1371/journal.ppat.1006278)
Supplement: S1 Guest Editor List — (PDF) [file ppat.1006278.s002.pdf]

PLOS Pathogens would like to thank all those who served as Guest Associate Editors in 2016:

Paul Ahlquist
David Allred
J. Alspaugh
Tim Anderson
Carlos Arias
Susan Baker
Henry Balfour
Mitchell Balish
Mark Banfield
Charles Bangham
Matthew Barber
Carolina Barillas-Mury
Peter Barry
Michael Beard
Vivian Bellofatto
Jeffrey Bergelson
David Bisaro
Samuel Black
Jesse Bloom
Sandrine Bourdoulous
Marcelo Bozza
Thomas Braciale
Paul Brindley
Igor Brodsky
David Brooks
James Brown
Barbara Burleigh
Edward Campbell
John Carr
Mark Carrington
Eric Cascales
Roberto Cattaneo
Matthew Chapman
Charles Chiu
Luka Čičin-Šain
Christine Clayton
Donald Coen
Jorn Coers
Marco Colonna
Inger Damon
Stephen Davies
Angus Dawe
Hilde de Reuse
Frank DeLeo
Neal DeLuca
Eric Delwart
Mahalia Desruisseaux
Darrell Desveaux
Daniel DiMaio
Roberto Docampo
Peter Dodds
Christian Drosten
Siobain Duffy
Ron Dzikowski
Catarina Eira
Najib El-Sayed
Markus Engstler
Luis Enjuanes
Lynn Enquist
Ananias Escalante
Roger Everett
Bart Everts
Bryce Falk
Mario Feldman
Katrina Forest
Matthew Frieman
Friedrich Frischknecht
Robert Garcea
Fernando Garcia-Arenal
Nisha Garg
Roberto Garofalo
Timothy Geary
Thomas Geisbert
Angie Gelli
Caroline Genco
N. Louise Glass
Britt Glaunsinger
Felicia Goodrum
Alexander Gorbalenya
Stephen Gottschalk
Scott Gray-Owen
Richard Grencis
Diane Griffin
Eduardo Groisman
Angelika Grundling
Haitao Guo
Linda Hazlett
Ekaterina Heldwein
Christopher Henry
Thomas Henry
Scott Hensley
Debroski Herbert
Ann Hill
Hans Hirsch
Stacy Horner
Michael Hsieh

Jianming Hu
Kelly Hughes
Christopher Hunter
Lars Hviid
Michael Imperiale
Javier Irazoqui
Francis Jiggins
Deirdre Joy
Isgouhi Kaloshian
Charu Kaushic
Alexander Khromykh
Karla Kirkegaard
Robyn Klein
Sabra Klein
Matty Knight
Peter Krell
Laurie Krug
Meta Kuehn
Krishan Kumar
Nicole La Gruta
Robert Lamb
Lewis Lanier
Wyndham Lathem
Michael Lattorff
Mansun Law
David Leib
Deborah Lenschow
Elena Levashina
Anice Lowen
Aron Lukacher
Shinji Makino
Richard Martin
Wendy Maury
Malcolm McConville
Rachel McLoughlin
W. Robert McMaster
Michael McVoy
Joan Mecsas
Xiang-Jin Meng
Lloyd Miller
Samuel Miller
Joachim Morschhäuser
Jeremy Mottram
Adrian Mountford
Christian Munz
Philip Murphy
Jim Nataro
William Navarre
Kirsten Nielsen
Mairi Noverr
Gabriel Nunez
Audrey Odom
David Ornelles
Nikolaus Osterrieder
Eric Oswald
Michael Otto
Annette Oxenius
James Paton
John Patton
James Paulson
Mark Peeples
Stanley Perlman
Sallie Permar
Nathan Peters
Beena Pillai
James Pipas
Richard Plemper
Mikhail Pletnikov
Alan Rickinson
Guus Rimmelzwaan
Amariliz Rivera
Michael Robek
Cliona M Rooney
Marilyn Roossinck
J. Rowe
Sarah Rowland-Jones
Charles Samuel
D. Scott Samuels
Rafael Sanjuán
Philip Santangelo
Peter Sarnow
Sara Sawyer
Mario Schelhaas
Stacey Schultz-Cherry
Thomas Schulz
Erwin Schurr
Martin Schwemmle
Bert Semler
Ganes Sen
Karol Sestak
Karl Seydel
Libo Shan
Robert Siliciano
João Silva
Larry Simpson
Arne Skorping
David Skurnik
Lynn Soong
Vanessa Sperandio
Peter Staeheli
Thilo Stehle
Boris Striepen
Lishan Su
Christopher Sullivan
George W. Sundin
Nicholas Talbot
Christoph Tang
Hengli Tang
Rita Tewari
Edward Thorp

Leann Tilley
David Tobin
Victor Torres
M. Stephen Trent
Emily Troemel
David Tscharke
Elaine Tuomanen
Paul Turner
Jude Uzonna
Akhil Vaidya
Jan van Kan
Steven Varga
Leda Vieira
Yuanchao Wang
Scott Weaver
Friedemann Weber
Leor Weinberger
Sandra Weller
Edward Wenger
Marvin Whiteley
Anna Whitfield
Kim Williamson
Patrick Wilson
Richard Wilson
Matthew Wolfgang
Daniel Wozniak
Qingfa Wu
Yan Xiang
George Yap
John Yates
Jacob Yount
Andrew Yurochko
Allan Zajac
Colby Zaph
Kai Zhang
Qijing Zhang
Zhi-Ming Zheng
Xueping Zhou
