# Supplementary material for: PLOS Pathogens 2016 Reviewer and Editorial Board Thank You
Source: PLoS Pathog. 2017 Mar 20;13(3):e1006278. doi: 10.1371/journal.ppat.1006278 (PMC5358882; doi:10.1371/journal.ppat.1006278)
Supplement: S1 Reviewer List — (PDF) [file ppat.1006278.s003.pdf]

PLOS Pathogens would like to thank all those who reviewed on behalf of the journal in 2016:

Alejandro Aballay
Ali Abdul-Sater
Laurent Abel
Allison Abendroth
Jonathan Abraham
Robert Abramovitch
Jacqueline Abranches
Hans Ackerman
Margaret Ackerman
Alvaro Acosta-Serrano
Luis Actis
Hervé Agaisse
Hector Aguilar
Patricia Aguilar
Sebastian Aguirre
Brian Ahmer
Jin-Hyun Ahn
Baki Akgül
Bungo Akiyoshi
Klaus Aktories
Pietro Alano
Jan Albert
Markus Albert
Anna Aldovini
Bree Aldridge
James Alexander
Martha Alexander-Miller
James Alfano
Juan Alfonzo
Holly Algood
Todd Allen
Allen Allen Miller
Igor Almeida
Francis Alonzo III
Michael Alpert
J. Alspaugh
Nihal Altan-Bonnet
Craig Altier
Neal Alto
Xavier Alvarez
James Alwine
Rama Amara
Gaya Amarasinghe
Carmen Amaro
Richard Ambinder
Zandrea Ambrose
Amal Amer
Rogiero Amino
Brian Amman
Linda Amos
Derk Amsen
Gregor Anderluh
John Andersen
Deborah Anderson
Paul Anderson
Richard A. Anderson
Wayne Anderson
David Andes
Christopher Andoniou
Bruno Andrade
Helene Andrews-Polymenis
Ourania Andrisani
Elliot Androphy
Serge Ankri
Aftab Ansari
Cristian Apetrei
Victor Appay
Grace Appiah
Judith Appleton
Jacques Archambault
Nancie Archin
Dawn Arnold
Michelle Arnold
Anna Arnqvist
Stefan Arold
David Aronoff
Sassan Asgari
David Asmuth
Sebastian Asurmendi
Marco Antonio Ataide
John Atkinson
Robert Atmar
Walter Atwood
Michelle Audsley
Victoria Auerbuch
Yossef Av-Gay
Panisadee Avirutnan
Jacob Ayers
Maria Ayllon
Abdu Azad

Nina Babel
Subash Babu
Michael Bachman
Steffen Backert
Michael Bader
Justin Bahl
Yong-Sun Bahn
Guangchun Bai
Susanne Bailer
Charles Bailey
Justin Bailey
Mark Bailey
Robert Baiocchi
Anna Bakardjiev
Brian Baker
David Baker
Susan Baker
Craig Baker-Austin
Guus Bakkeren
Siddharth Balachandran
Kithiganahalli Balaji
Jimmy Ballard
Bruce Bamber
Christianne Bandeira de Melo
Bruce Banfield
Mark Banfield
Charles Bangham
James Bangs
Lawrence Banks
Xiaodong Bao
Xiaoyong Bao
Benoît Barbeau
Daniel Barber
Winfried Barchet
Wendy Barclay
Stephen Barenkamp
Sailen Barik
Carolina Barillas-Mury
Bridget Barker
Edward Barker
Eric Barklis
Ellie Barnes
Dan Barouch
John Barr
Simon Barratt-Boyes
Alan Barrett
Michael Barrett
Clifton Barry
Peter Barry
Ralf Bartenschlager
Richard Bartfai
David Barton
Thomas Baumert
Nicole Baumgarth
Gillian Beamer
David Beasley
Stephan Becker
J. David Beckham
Sammy Bedoui
Michael Beekes
James Beeson
Samuel Behar
John Bell
George Belov
Graham Belsham
Gabrielle Belz
Jorge Benach
Javier Benavente
José Bengoechea
Monsef Benkirane
Andrew Bent
Stephen Bentley
William Bentley
Mikkel Bentzon-Tilia
Roland Benz
Rance Berg
Jeffrey Bergelson
Cornelia Bergmann
Andreas Bergthaler
Vincent Beringue
Arnold Berk
Ben Berkhout
Kristen Bernard
Thomas Bernhardt
Maud Bernoux
Lionel Berthoux
Antonio Bertoletti
Brent Berwin
Alain Beschin
Aaron Best
Sonja Best
Dany Beste
Sébastien Besteiro
Michael Betts
Jim Beynon
Alok Bhattacharya
Roman Biek
Brigitte Biesinger-Zwosta
Elaine Bignell
Paul Birch
Christine Biron
Markus Bischoff

Wilbert Bitter
Lars Björck
Niklas Björkström
Dieter Blaas
Samuel Black
Ira Blader
Carol Blair
Joshua Blakeslee
Guillaume Blanc
Stéphane Blanc
Anne Blangy
Joel Blankson
David Blehert
Catherine Blish
James Bliska
Joseph Bliss
David Bloom
Jesse Bloom
Thomas Bobik
Justin Boddey
Jochen Bodem
Rogier Bodewes
Jason Bodily
David Boehr
Dusan Bogunovic
Jennifer Bomberger
Morgane Bomsel
Stéphane Bonacorsi
Matteo Bonazzi
Marcelo Bonini
Marc Bonten
Adrianus Boon
Andre Boonstra
Seth Bordenstein
Kathleen Boris-Lawrie
Eli Boritz
Bradley Borlee
Peter Boross
Karina Bortoluci
Irene Bosch
Jeff Bose
Steven Bosinger
Katharine Bossart
Cyrille Botté
Michael Bouchard
Thierry Boulinier
Kostas Bourtzis
Bernard Bouteille
Freddy Boutrot
Thomas Bowden
Dawn Bowdish
Laura Boykin
Michelle Boyle
Thomas Braciale
Andrea Branch
Curtis Brandt
Ignacio Bravo
Klaus Brehm
Jason Brenchley
Greg Brennan
Michael Brennan
Richard Brennan
Stephane Bressanelli
James Brewer
Ian Brierley
John Briggs
Volker Briken
Paul Brindley
Melanie Brinkmann
Shaun Brinsmade
Catherine Brissette
Robert Britton
Jennifer Brodbelt
Priscille Brodin
Igor Brodsky
Heike Broetz-Oesterhelt
Chris Brooke
David Brooks
Roland Brosch
Laurent Brossay
Gordon Brown
Mark Brown
Michael Brown
Richard Brown
Jeremy Brownlie
Petr Broz
Katja Brückner
John Brumell
Wolfram Brune
Frédéric Brunner
Volker Bruss
Yenan Bryceson
Juliane Bubeck Wardenburg
Ursula Buchholz
Nicolas Buchon
Christopher Buck
Bryce Buddle
James Bull
Peter Bull
Lee Bulla
Dirk Bumann
Christina Burch

Tricia Burdo
Ian Burgess
Barbara Burleigh
Robin Bush
Kathryn Bushley
Noah Butler
Oleg Butovsky
Mariana Byndloss
Didier Cabanes
Ken Cadwell
Guohong Cai
James Cai
Melissa Caimano
Jennifer Cameron
Mary Cameron
Andrew Camilli
Gabriella Campadelli-Fiume
Anthony Campagnari
David Campbell
Edward Campbell
Kenneth Campellone
Oscar Campetella
Samuel Campos
Antonio Campos-Neto
Bruno Canard
Michael Caparon
Patrizia Caposio
Massimo Caputi
Nicholas Carbonetti
Rhonda Cardin
Jonathan Carlson
Daniel Carr
John Carr
Agostinho Carvalho
Josep Casadesús
Arturo Casadevall
Climent Casals-Pascual
Jim Casanova
John Casey
James Cassat
Maria Cassera
Joaquín Castilla
Luis Castillo
Clayton Caswell
Marta Catalfamo
Maria Catanese
Roberto Cattaneo
Amy Caudy
Byron Caughey
Linda Cauley
Jen Cavet
Jean Celli
Ethel Cesarman
Kris Chadee
Ann Chahroudi
Wengang Chai
Gary Chan
John Chan
Bala Chandran
Kartik Chandran
Theresa Chang
Yuan Chang
Rudragouda Channappanavar
Saurabh Chattopadhyay
Esteban Chaves-Olarte
Ian Cheeseman
Karthi Chellappa
Benjamin Chen
Bing Chen
Hao Chen
Mei-Ru Chen
Yin Chen
Zhixiang Chen
Alexandre Chenal
Alan Cheng
Genhong Cheng
Gong Cheng
Cecilia Cheng-Mayer
Peter Cherepanov
Bruce Chesebro
G. Chinnadurai
James Chodosh
Kyung Choi
Nicolas Chomont
Yi-Ying Chou
Neil Christensen
Peter Christie
George Christophides
Justin Jang Hann Chu
Konstantin Chumakov
Amy Chung
Chin Ha Chung
Michael Ciancanelli
Andrea Cimarelli
Vincenzo Ciminale
Paola Cinque
Paul Clapham
Thomas Clarke
François Clavel
Julie Claycomb
Christine Clayton
Rollie Clem

Daniel Clemens
Janice Clements
Anna Cliffe
Gitta Coaker
Aurélie Cobat
Steven Cobb
Sarah Cobey
Ian Cockburn
Donald Coen
Tom Coenye
Jorn Coers
Kenneth Coggeshall
Jeffrey Cohen
Taylor Cohen
Randall Cohrs
James Collins
Kathleen Collins
Ronald Collman
Alan Collmer
Marco Colonna
Tonya Colpitts
Iñaki Comas
Brian Conlon
Elizabeth Connick
John Connor
Heather Conti
James Conway
Klaus Conzelmann
Gregory Cook
Roland Cooper
Lawrence Corey
Stephania Cormier
Mauro Cortez
Davide Corti
Francois-Loic Cosset
Peggy Cotter
Sheena Cotter
Barbara Coulson
Kevin Couper
Mathieu Coureuil
Harry Courtney
Anna Coussens
Timothy Cover
Benjamin Cowling
Andrea Cox
Carolyn Coyne
Alister Craig
Alison Criss
Ileana Cristea
George Cross
Robert Cross
Sean Crosson
Shane Crotty
Nicholas Croucher
James Crowe Jr.
James Culver
Edecio Cunha-Neto
Nik Cunniffe
Adam Cunningham
Anthony Cunningham
Aubrey Cunnington
Tom Cupedo
Mamadou Daffé
Wassim Daher
Wei Dai
Tina Dalianis
Marc Dalod
Satya Dandekar
Rachel Daniels
Yael Danin-Poleg
Charles Dann III
Fabien Darfeuille
Siddhartha Das
Sandip Datta
Miles Davenport
John David
Stephen Davies
Dana Davis
Richard Davis
Angus Dawe
Suzanne Dawid
Scott Dawson
Cheryl Day
Tim Day
Marco De Andrea
Z. Wilhelm de Beer
Rob De Boer
Luiz Pedro de Carvalho
Christopher de Graffenried
Sybren de Hoog
Harry De Koning
Hilde de Reuse
Rik de Swart
Nicola Decaro
Steven Deeks
George Deepe Jr.
Victor Defilippis
Ala-Eddine Deghmane
Christoph Dehio
Frank Deleo
Gustavo Delhon
Graham Dellaire

Neal Deluca
Qiang Deng
Mark Denison
Cynthia Derdeyn
Petra Dersch
Sanjay Desai
Albert Descoteaux
Darrell Desveaux
Corrella Detweiler
Veena Devi Ganeshan
Stephanie Dewitte-Orr
Nolwenn Dheilly
Antonio Di Pietro
Felipe Diaz-Griffero
Martin Dickman
Lars Dietrich
Ralf Dietzgen
Ivan Dikic
Adler Dillman
Marcus Dillon
Daniel Dimaio
Maria D'Império Lima
Charles Dinarello
Colette Dissous
Maziar Divangahi
Richard Dix
Linda Dixon
Armin Djamei
David Dockrell
Hazel Dockrell
Anna Dongari-Bagtzoglou
Sheila Donnelly
Nicole Donofrio
John Doorbar
Katie Doores
Anca Dorhoi
Pedro D'Orleans-Juste
Katerina Dorovini-Zis
Daolong Dou
Vincent Doublet
Paschalis-Thomas Doulias
Jan Felix Drexler
Adam Driks
Christian Drosten
Heidi Drummer
Jean Dubuisson
Gytis Dudas
Jaquelin Dudley
Patrick Duffy
Siobain Duffy
Roy Duncan
Stephen Duncan
Sarah Dunstan
W. Paul Duprex
Madeleine Durbeej
Joan Durbin
Olivier Duron
Michael Duszenko
Rebecca Dutch
Malcolm Duthie
Bernadette Dutia
Jonathan Dworkin
Kevin Dybvig
Ron Dzikowski
Andrew Easton
Gregory Ebel
Hideki Ebihara
Philip Eckhoff
Lars Eckmann
Mariola Edelmann
Brian Edelson
Mira Edgerton
Stacey Efstathiou
Rebecca Eisen
Wolfgang Eisenreich
Farid El Kasmi
Karen Elkins
Ali Ellebedy
Craig Ellermeier
Tim Elliott
Jeff Ellis
Stephane Emiliani
Susanne Engelmann
David Engman
Markus Engstler
Luis Enjuanes
Alexander Ensminger
Armin Ensser
Marc Erhardt
Joel Ernst
Jacob Estes
Jay Evans
Matthew Evans
Roger Everett
David Everly
Jonathan Ewbank
Katherine Excoffon
Oliver Fackler
Abeer Fadda
Neil Fairweather
Bryce Falk
Maria Fallman

Ann Fallon
Padraic Fallon
Paul J. Farrell
Nicolas Fasel
Ariberto Fassati
Bruno Favery
Rachel Fearns
Michael Federle
Anthony Fehr
Heinz Feldmann
Simon Fellous
Ming-Guang Feng
Zongdi Feng
Kevin Fennelly
Sara Ferrando-Martinez
Dominique Ferrandon
Guido Ferrari
Matthew Ferrari
Marcelo Ferreira
Richard Ferrero
Paul Fey
Helmut Fickenscher
Paul Fidel Jr.
David Fidock
Mark Field
Kenneth Fields
Melania Figueroa
Paul Fine
Stefan Finke
Natosha Finley
Andres Finzi
Matthias Fischer
Nicole Fischer
Wolfgang Fischer
Matthew Fisher
J. Bert Flanegan
Suzanne Fleiszig
Michelle Flenniken
Helen Fletcher
Luise Florin
Andres Floto
Ervin Fodor
Denise Fonseca
Krystal Fontaine
Mandy Ford
Gianluigi Forloni
J. Forrest
Donald Forthal
Louis-Charles Fortier
Elizabeth Fortunato
Timothy Foster
Woodbridge Foster
Howard Fox
Betsy Foxman
Nathalie Franc
Genoveffa Franchini
Matthew Francis
Daniel Frank
Alan Frankel
Gad Frankel
Lori Frappier
Christophe Fraser
Christopher Fraser
Iain Fraser
James Fraser
John Frater
Eric Freed
Nancy Freitag
Jeffrey Frelinger
Lisa Frenkel
Manuel Fresno
Ute Frevert
Matthew Frieman
Teresa Frisan
Friedrich Frischknecht
Jörg Fritz
Simon Frost
Marc Fuchs
Takashi Fujita
Jennifer Furin
Julia G. Prado
Gülsah Gabriel
Jorge Galan
David Galbraith
Mary Galinski
Philippe Gallay
Giorgio Gallinella
Amit Gal-On
Benoît Gamain
Yunn-Hwen Gan
Soren Gantt
Tomas Ganz
Laurent Gapin
Robert Garcea
Fernando Garcia-Arenal
Mariano Garcia-Blanco
Dominique Garcin
Matt Gardner
Abhishek Garg
Robert Garry
Danielle Garsin
Anne Gatignol

Yves Gaudin
Timothy Geary
Adam Geballe
Alma Gedvilaite
Adam Gehring
Teunis Geijtenbeek
Thomas Geisbert
Nelson Gekara
Christophe Geldmacher
Annemieke Geluk
Elke Genersch
Brian Gentry
Nidhi Gera
Volker Gerdts
Ralf Gerhard
Andrew Gewirtz
Benjamin Gewurz
Veikko Geyer
Mahmoud Ghannoum
Elodie Ghedin
Frank Gheradini
Paul Giacomin
Chou-Zen Giam
Sara Gianella
Wade Gibson
Mark Gijzen
Tim Gilberger
Clement Gilbert
Ian Gilbert
Geraldine Gillespie
Michael Ginger
Stephen Girardin
Britt Glaunsinger
Cynthia Gleason
H. Charles Godfray
Dale Godfrey
Paul Goepfert
Grazielle Goes
Jon Goguen
Daniel Goldberg
Joanna Goldberg
Tony Goldberg
William Goldman
Dasantila Golemi-Kotra
Kenneth Gollob
Tatyana Golovkina
Mark Gomelsky
James Gomez
Fernando Gonzalez-Candelas
Mercedes Gonzalez-Juarrero
Ana Gonzalez-Reiche
Elena Gonzalez-Rey
Steven Goodman
Heidi Goodrich-Blair
Alexander Gorbalenya
Stephen Gordon
Jorg Goronzy
Heinrich Gottlinger
Stephen Gottschalk
Eva Gottwein
Marie-Lise Gougeon
Mark Goulian
Richard Gourse
Shubha Govind
Yonatan Grad
Sheila V. Graham
John Grainger
Arash Grakoui
Michael Gray
Scott Gray-Owen
Urs Greber
William Green
Benjamin Greenbaum
David Greenberg
Harry Greenberg
Robert Greenberg
Richard Grencis
Christoph Grevelding
Paul Griffiths
Irina Grigorova
Sergio Grinstein
Carol Gross
Christoph Grunau
Adam Grundhoff
John Guatelli
Nancy Guillen
Suryaram Gummuluru
John Gunn
Haitao Guo
Zhongxin Guo
Nishith Gupta
Andrea Gust
David Haake
Rainer Haas
Abderrahman Hachani
Ted Hackstadt
Maria Hadjifrangiskou
Julius Clemence Hafalla
Alexander Hahn
Nancy Haigwood
Stephen Hajduk
Mohamed Hakimi

Rebecca Hall
Roy Hall
Otto Haller
Marc Halushka
Marie-Louise Hammarskjold
Tansy Hammarton
Brian Hammer
Gianna Hammer
Neal Hammer
Sven Hammerschmidt
Wolfgang Hammerschmidt
Katie Hampson
Yiping Han
Meaghan Hancock
Lars Hangartner
Tomas Hanke
Diana Hansen
Ashraful Haque
Philip Hardwidge
Edward Harhaj
William Harnett
Elizabeth Harrington
Reuben Harris
Steven Harris
Tajie Harris
Joe Harrison
Stephen Harrison
Dominik Hartl
Elizabeth Hartland
Rune Hartmann
Jonathan Harton
Ronald Harty
Richard Harvey
Eric Harvill
Hassan Hashimi
Philip Hastings
Christof Hauck
Vasili Hauryliuk
Christopher Hayes
Barton Haynes
S. Hayward
Biao He
Cynthia He
Ya-Wen He
Yongqun He
William Heath
Nicholas Heaton
David Heckel
John Hegarty
Adrian Hehl
Simon Heilbronner
Manfred Heinlein
Krista Heinonen
Franz Heinz
Robert Heinzen
Zdenek Hel
Sophie Helaine
Ekaterina Heldwein
Ari Helenius
John Helmann
Helena Helmby
Andrew Henderson
Ian Henderson
Jeffrey Henderson
Robert Hendricks
Birgitta Henriques-Normark
Cesar Henriquez-Camacho
Thomas Henry
Scott Hensley
Joshua Herbeck
Georges Herbein
Debroski Herbert
Ruben Hernandez-Alcoceba
Betsy Herold
Susanne Herold
Johannes Herrmann
Tomer Hertz
Mark Herzberg
Volker Heussler
Heather Hickman
Yasufumi Hikichi
Hubert Hilbi
Rolf Hilgenfeld
Alison Hill
Ann Hill
Darryl Hill
N. Luisa Hiller
Bradley Hillman
Alec Hirsch
Vanessa Hirsch
Robert Hirt
Julian Hiscox
Ya-Chi Ho
Lucas Hoffman
Alexander Hoffmann
Ary Hoffmann
Michael Hofreiter
Monica Höfte
Deborah Hogan
James Hogle
Kristin Hogquist
Tobias Hohl

Michael Holbrook
Matthew Holden
Lindy Holden-Dye
Anthony Holder
Edward Holmes
Lauren Hook
Magnus Hook
Martin Horn
Mathias Hornef
Stacy Horner
William Horsnell
Alexander Horswill
Curt Horvath
Fanjian Hou
Ross Houston
Jonathan Howard
Michael Howard
Benjamin Howden
Peter Howley
James Hoxie
Nathanael Hoze
Michael Hsieh
Guochang Hu
Jianming Hu
Xiaoyu Hu
I-Chueh Huang
Bernhard Hube
Philippe Huber
Stephan Huehn
David Hughes
Grant Hughes
Molly Hughes
Stephen Hughes
Jon Huibregtse
Scot Hulbert
Scott Hultgren
Ian Humphreys
Peter Hunt
Christopher Hunter
Eric Hunter
Ryan Hunter
Christopher Huston
Anna Huttenlocher
Lars Hviid
Seungmin Hwang
Matteo Iannacone
Jose Ibeas
Ashraf Ibrahim
Alexander Idnurm
Jean-Luc Imler
Michael Imperiale
Hanne Ingmer
Roger Innes
Naohiro Inohara
Ronald Iorio
Javier Irazoqui
David Isenman
Antonella Isgro
Masanori Isogawa
Ivaylo Ivanov
Astrid Iversen
Akiko Iwasaki
Yasumasa Iwatani
Luis Izquierdo
Yoshihiro Izumiya
William Ja
Mary Ann Jabra-Rizk
Robert Jackson
William Jackson
Francoise Jacob-Dubuisson
Marcelo Jacobs-Lorena
Prasanna Jagannathan
Sanjay Jain
Stephen Jameson
Eric Jan
Sung Key Jang
Dragana Jankovic
Edith Janssen
Christian Janzen
Ted Jardetzky
Babak Javid
Ronald Javier
Michael Jeger
Jae-Wook Jeong
Ann Jerse
Holger Jeske
Sudhakar Jha
Daohong Jiang
Wen Jiang
Zhengfan Jiang
Dong-Yan Jin
Hailing Jin
Christian C Jobin
Ludger Johannes
Eric Johannsen
Chandy John
Gareth John
Susan John
Pål Johnsen
David Johnson
Jack Johnson
Karyn Johnson

Kyle Johnson
R. Paul Johnson
Reed Johnson
Welkin Johnson
Calum Johnston
Simon Johnston
Clare Jolly
Emmitt Jolly
Anthony Jones
Brad Jones
Clinton Jones
Stipan Jonjic
Colleen Jonsson
Simone Joosten
Christine Josenhans
Sarah Joseph
Sunil Joshi
Laure Journet
Howard Judelson
Jae Jung
Jae U. Jung
Isabelle Jupin
Sheryl Justice
Aras Kadioglu
Jonathan Kagan
J. Michelle Kahlenberg
Murali Kaja
Rob Kalejta
Vipin Kalia
Ulrich Kalinke
Shaden Kamhawi
Jeremy Kamil
Sophien Kamoun
Patricia Kane
Seogchan Kang
Mari Kannagi
Maria Kaparakis-Liaskos
Ronan Kapetanovic
Stefan Kappe
John Karijolich
Jonathan Karn
Stephanie Karst
Fatah Kashanchi
Dennis Kasper
Sudhir Kasturi
Aris Katzourakis
Paul Kaufman
Radhey Kaushik
Annemieke Kavelaars
Thomas Kawula
Kenneth Kaye
Paul Kaye
Barbara Kazmierczak
Leslie Kean
Joseph Keane
Mary Kearney
Karen Keddy
Katherine Kedzierska
Brandon Keele
Thomas Kehl-Fie
Marcus Kehrli
Nancy Keller
John Kelly
Eric Kemen
Scott Kennedy
Scott Kenney
Shannon Kenney
Brendan Kenny
Stephen Kent
Florian Kern
Vineet Kewalramani
Nemat Keyhani
Imtiaz Khan
Rajiv Khanna
Jeff Kieft
Margaret Kielian
Tammy Kielian
Gustavo Kijak
Choel Kim
Dennis Kim
Kwang Kim
Peter Kim
Peter Kima
Anthony Kincaid
Paul Kinchington
Irah King
Kayla King
Robert Kingsley
John Kirby
Frank Kirchhoff
Natalia Kirienko
Karla Kirkegaard
Uday Kishore
Scott Kitchen
Morten Kjos
P.J. Klasse
Nichole Klatt
Florian Klein
Robyn Klein
Michael Klemba
Paul Klenerman
Kimberly Kline

Aloysius Klingelhutz
Jonas Klingstrom
Karl Klose
David Knipe
Leigh Knodler
Toshihide Kobayashi
Gary Kobinger
Lester Kobzik
David Koelle
Katia Koelle
Alain Kohl
Beverly Koller
Jay Kolls
Dennis Kolson
Zhaosheng Kong
Michael Koomey
Bette Korber
Hardy Kornfeld
Nicole Koropatkin
Sergei Kosakovsky Pond
Anita Koshy
Konstantin Kousoulas
Roger Kouyos
Susan Kovats
Timothy Kowalik
Yoshio Koyanagi
Christine Kozak
Florian Krammer
Sven Krappmann
Duncan Krause
Karl Heinz Krause
Kirsten Krause
Philip Krause
Eric Kremer
Jan Kreuze
Sanjeev Krishna
Krister Kristensson
Thomas Kristie
Abby Kroken
James Kronstad
Laurie Krug
Robert Krug
Eric Krukonis
Claude Krummenacher
Per Kryger
Urszula Krzych
Ersheng Kuang
Karl Kuchler
Dmitri Kudryashov
Meta Kuehn
Thomas Kufer
Jon Kull
Carol Kumamoto
Ashok Kumar
Nirbhay Kumar
Sanjai Kumar
Rei-Lin Kuo
Inari Kursula
Olaf Kutsch
Marc Kvansakul
Mamuka Kvaratskhelia
Peter Kwong
Tina Kyndt
Juan Laclette
D. Borden Lacy
Frank Lafont
William Lafuse
Laurel Lagenaur
Michael Lagunoff
Thomas Lahaye
Erh-Min Lai
Lou Laimins
Seema Lakdawala
Jean-François Laliberté
Don Lamb
Tracey Lamb
Richard Lamont
Ke Lan
Meytal Landau
Nathaniel Landau
Alan Landay
Scott Landfear
Santo Landolfo
Ryan Langlois
William Langridge
Gordon Langsley
Joseli Lannes-Vieira
Natalia Lapteva
Christopher Larock
Jean-Paul Latge
Wyndham Lathem
Georg Lauer
Renia Laurent
Grégoire Lauvau
Thomas Lavstsen
Mansun Law
Matthew Lawrenz
Phillip Lawyer
Laura Layland
Beth Lazazzera
Helen Lazear
Brian Lazzaro

Roger Le Grand
Jacques Le Pendu
Karine Le Roch
Marc-Henri Lebrun
Marc Lecuit
Michael Lederman
Chia Lee
Nara Lee
Nelson Lee
Soo Chan Lee
Yong-Hwan Lee
Ann Leen
Fabian Leendertz
Kevin Legge
Giuseppe Legname
Adele Lehane
Paul Lehner
David Leib
Salome Leibundgut-Landmann
Veerle Lejon
Isabelle Lemasson
Philippe Lemey
Katherine Lemon
José Lemos
Wayne Lencer
Deborah Lenschow
Laurel Lenz
Paul Leon
Julien Lescar
Gabriel Leventhal
Stuart Levitz
Shawn Lewenza
Sharon Lewin
Amanda Lewis
George Lewis
Jennifer Lewis
Victor Leyva-Grado
Chengwen Li
Chris Li
Jonathan Li
Kui Li
Qingsheng Li
Xiao-Dong Li
Xin Li
Zhenghe Li
Ziyin Li
Chen Liang
Jinling Liao
Paul Liberman
Mathias Lichterfeld
Tami Lieberman
Egil Lien
Petros Ligoxygakis
Aaron Lin
Rongtuan Lin
Xin Lin
Zhen Lin
Gunnar Lindahl
Cecilia Lindestam Arlehamn
Steven Lindow
Jaisri Lingappa
Dirk Linke
Michail Lionakis
Roger Lippé
Botao Liu
Cindy Liu
Fenyong Liu
George Liu
Haoping Liu
Jinhua Liu
Yule Liu
Marcela Lizano
Manuel Llano
Clare Lloyd
James Lloyd-Smith
Chu-Fang Lo
Nicolas Locker
Shawn Lockhart
Daniel Loeb
Martin Loechelt
Volker Lohmann
James Lok
James Lokensgard
Richard Lo-Man
Heather Long
Ulisses Lopes
Leila Lopes-Bezerra
Carolina Lopez
Miguel López-Botet
Juan Jose Lopez-Moya
Jose Lopez-Ribot
Michael Lorenz
Alex Loukas
Sebastian Lourido
Philip Loverde
Anice Lowen
Franklin Lowy
Chun Lu
Shan Lu
Yuanan Lu
Jeremy Luban
Stephan Ludwig

Karolin Luger
Anja Lührmann
Julius Lukes
Joseph Luna
Joachim Lupberger
C. Patrick Lusk
Paolo Lusso
Sara Lustigman
Hinh Ly
Gareth Lycett
Samantha Lycett
Rebecca Lynch
Vincent Lynch
Jiyan Ma
Li-Jun Ma
Andrew MacDonald
Fabiana Machado
Matthias Machner
Alberto Macho
Jason Mackenzie
Erich Mackow
Juliette Madan
Kelly Magalhaes
Marco Magalhaes
Diogo Magnani
Katharine Magor
Suresh Mahalingam
Jitendra Maharana
Renaud Mahieux
Berenike Maier
Rick Maizels
George Makhatadze
Frank Maldarelli
Pawan Malhotra
Harmit Malik
Richard Malley
Kevin Maloy
Maksim Mamonkin
Mark Mandel
Nicolas Manel
Nicholas Maness
Balaji Manicassamy
Adhar Manna
Dan Manor
Susanna Manrubia
Louis Mansky
Jean Manson
Nicholas Mantis
Maria Marco
Joseph Marcotrigiano
David Margolis
Leonid Margolis
Chelsea Marie
Alberto Marina
Kevin Maringer
Roy Mariuzza
Varpu Marjomaki
Brian Mark
Eric Marois
Joao Marques
Benjamin Marsland
Matthias Marti
Malcolm Martin
Alberto Martin
Elena Martinelli
Javier Martinez-Picado
Encarnacion Martinez-Salas
Luis Martínez-Sobrido
Francisco Marty
Andrea Marzi
Hideaki Maseda
Pascal Mäser
Kevin Mason
Paul Masters
Maria Masucci
Anuja Mathew
Masao Matsuoka
Joseph Mattapallil
Keith Matthews
Stephen Matthews
Kai Matuschewski
Felix Mauch
Anthony Maurelli
Wendy Maury
Josef Mautner
Eric May
Christoph Mayer
Katrin Mayer-Barber
Shonna McBride
Dennis McCance
James McCarthy
Douglas McCarty
Mark McClain
Malcolm McConville
A. Louise McCormick
Craig McCormick
Richard McCulloch
Joseph McCune
David McDermott
Christopher McDevitt
Christine McDonald
Megan McDonald

Sarah McDonald
Geoffrey McFadden
Maureen McGargill
Gerald McInerney
Kevin McIver
Derek McKay
Debbie McKenzie
Valerie McKenzie
Kai McKinstry
John McLauchlan
Margaret McLaughlin-Drubin
Diane McMahon-Pratt
Janet McNicholl
Neil McRoberts
Stephen McSorley
Michael McVoy
Hamish McWilliam
Eva Medina
Andrew Mehle
Jeffery Meier
Annemarie Meijer
Markus Meissner
Thomas Melendy
Gregory Melikyan
Craig Mello
Vineet Menachery
Jason Mercer
Tod Merkel
Janet Mertz
Mark Mescher
Enrique Mesri
Ilhem Messaoudi
William Messer
Martin Messerle
Dennis Metzger
Austin Meyer
Thomas Meyer
José Meyer-Fernandes
Edward Miao
Shulamit Michaeli
Thomas Michiels
Martin Middendorf
Sandra Milasta
Christopher Miller
Jeffrey Miller
Louis Miller
Matthew Miller
Samuel Miller
William Miller
Gregg Milligan
James Mills
Kingston Mills
Joel Milner
Booki Min
Michael Mina
Mike Minnick
Philip Minor
Chad Mire
Prashant Mishra
Dominique Missiakas
Guillaume Mitta
Makoto Miyata
Joseph Mizgerd
Valerie Mizrahi
Mahtab Moayeri
Yorgo Modis
Robert Modlin
Ian Mohr
Antonio Molina
Malcolm Molyneux
Mario Mondelli
David Montefiori
Branch Moody
Cary Moody
M. Moody
Christiane Moog
John Moore
Margo Moore
Martin Moore
Penny Moore
Tom Moore
Nathaniel Moorman
Ann Moormann
Guido Mora
Trevor Moraes
Rodrigo Morales
Thomas Moran
Gonzalo Moratorio
Dana Mordue
Jean-Benoît Morel
Edgardo Moreno
Silvia Moreno
Antonio Moretti
Iain Morgan
Robin Morgan
Enrique Moriones
Renato Morona
Craig Morrell
James Morris
Lynn Morris
Lynda Morrison
Sherie Morrison

Thomas Morrison
Joachim Morschhäuser
Jose Maria Alvarez Mosig
Bernard Moss
Paul Moss
Serge Mostowy
Bianca Mothe
Walther Mothes
Jeremy Mottram
Scott Moye-Rowley
David Moyes
Mary Beth Mudgett
Elke Muehlberger
Anne Mueller
Christian Muenz
Elke Mühlberger
Takafumi Mukaihara
Shaeri Mukherjee
Caitlin Mullarkey
Yves Muller
Barbara Müller
Marcel Müller
Michaela Müller-Trutwin
David Mullins
Matthew Mulvey
Jan Munch
Joshua Munger
Sean Munro
Vincent Munster
Christian Munz
Takayuki Murata
Elizabeth Murchison
Pablo Murcia
Eain Murphy
Kenneth Murphy
Michael Murphy
Barbara Murray
Peter Murray
Susan Murray
Michael Murtaugh
Lawrence Myers
Peter Myler
Kevin Myles
Eleftherios Mylonakis
Indira Mysorekar
Raffael Nachbagauer
Hiroki Nagai
Julian Naglik
Moon Nahm
Meera Nair
Helder Nakaya
Keiichi Namba
Francis Nano
Naweed Naqvi
Mohandas Narla
Michael Nassal
Jim Nataro
Sheila Nathan
Wiederhold Nathan
Lishomwa Ndhlovu
Richard Neher
Matthew Neiditch
Stuart Neil
Frank Neipel
David Nelson
Martha Nelson
Richard Nelson
Glen Nemerow
Donna Neumann
Gabriele Neumann
Michael Nevels
Cedric Neveu
Mari-Anne Newman
Hayley Newton
Irene Newton
Robert Newton
Olivier Neyrolles
Max Nibert
Anthony Nicola
Kirsten Nielsen
Per Nielsen
Stefan Niemann
Michael Niepmann
Stefan Niewiesk
Fangkun Ning
Marc Nishimura
Yorihiro Nishimura
Victor Nizet
Angela Nobbs
Nicholas Noinaj
Tony Nolan
Nobuhiko Nomura
Romolo Nonno
Steven Norris
Isabel Novella
Mairi Noverr
Martin Nowak
Tomoyoshi Nozaki
Ulrich Nübel
Torsten Nuernberger
Gabriel Nunez
Gerard Nuovo

Jennifer Nyborg
Per-Georg Nyholm
Susanne Nylen
Richard O’Connell
Joshua Obar
Meagan O'Brien
David O'Brochta
Christine O'Connor
David O'Connor
Una O'Doherty
Audrey Odom
Gilad Ofek
James O'Gara
Kristen Ogden
Marco Oggioni
Julia Oh
Taro Ohkawa
Akinyemi Ojesina
Hiroaki Okamoto
Gene Olinger
Martin Olivier
Akira Ono
Marcel Ooms
David Ornelles
Kim Orth
Andrew Osborne
Campetella Oscar
Maghnus O'Seaghdha
Nir Osherov
Taku Oshima
Mario Ostrowski
George O'Toole
Karen Ottemann
Michael Otto
Marc Ouellette
Julie Overbaugh
Annette Oxenius
Slobodan Paessler
Antonio Pagan
Patricia Paglini-Oliva
Anand Pai
Emil Pai
Mirko Paiardini
Mariya Pakharukova
Gustavo Palacios
Nades Palaniyar
Mark Pallansch
Mark Pallen
Massimo Palmarini
Søren Paludan
Franck Panabieres
Ivona Pandrea
Ralph Panstruga
Giuseppe Pantaleo
Julien Papaix
F. Nina Papavasiliou
Venizelos Papayannopoulos
Lars Pape
Jason Papin
Alexander Paredez
Leslie Parent
Andrew Park
Geun Woo Park
Lisa Parker
Griffith D. Parks
Francisco Parra
Colin Parrish
Chris Parsons
Sally Partridge
Jo-Ann Passmore
Vinay Pathak
John Patton
Nicole Pavio
Eric Pearlman
Roger Pechous
Scott Peck
R. Peebles Jr.
Mark Peeples
Olve Peersen
J.S. Malik Peiris
Philip Pellett
Jose Penades
Miguel Penalva
Carlos Penha-Goncalves
Caitlin Pepperell
Marta Perego
Mercio Pereiraperrin
Rushika Perera
Damian Perez Mazliah
Neil Perkins
David Perlin
Stanley Perlman
Steven Perlman
Sallie Permar
Carlo Federico Perno
Matthieu Perreau
Jean-Pierre Perreault
Deborah Persaud
B. Matija Peterlin
Brian Peters
Georg Peters
Nathan Peters

Townsend Peterson
Constantinos Petrovas
Melinda Pettigrew
Michael Pfaller
Sebastien Pfeffer
Julie Pfeiffer
Jennifer Philips
Michael Phillips
Mathieu Picardeau
Andreas Pichlmair
Raymond Pickles
Giovanni Piedimonte
Susan Pierce
Corné Pieterse
Thomas Pietschmann
Vincent Piguet
Gorben Pijlman
David Pintel
Abraham Pinter
James Pipas
Claudine Pique
Laura Pirisinu
Javier Pizarro-Cerda
Bodo Plachter
Magdalena Plebanski
Richard Plemper
Alexander Ploss
Maurizio Pocchiari
Eric Poeschla
Judit Pogany
Stefan Pöhlmann
Alessandra Polissi
Stephen Polyak
Liza Pon
Carolina Poncini
Mikhail Pooggin
Bert Poolman
Art Poon
Michel Popoff
Filippos Porichis
Owen Pornillos
Miriam Postan
Michael Potchen
Jan Potempa
Michael Povelones
Rafael Prados-Rosales
Reinhild Prange
Nemani Prasadarao
Gerd Prehna
Gail Preston
Peter Prevelige
Michael Price
Mark Prichard
Suzette Priola
Gerardo Priotto
Martin Prlic
Read Pukkila-Worley
Bali Pulendran
Tal Pupko
Georgiana Purdy
Michael Purdy
Dohun Pyeon
Feng Qu
Janet Quinn
Kylie Quinn
Vincent Racaniello
Stephen Rader
Sylvain Raffaele
Manuela Raffatellu
Erik Ragsdale
Murugesan Rajaram
Ricardo Rajsbaum
Glenn Rall
Katherine Ralston
Sanjay Ram
Srinivasan Ramakrishnan
Sasirekha Ramani
Juan David Ramirez
J.C. Ramos
Richard Randall
Troy Randall
Felix Randow
Stefanie Ranf
Hilary Ranson
A.L.N. Rao
Jayne Raper
Jay Rappaport
Rino Rappuoli
Joachim Rassow
Poonam Rath
John Rathjen
Pradipsinh Rathod
Ranjit Ray
Stuart Ray
Brandon Razooky
Fabio Re
Laurie Read
Patrick Reading
Leslie Real
Michael Reese
R. Keith Reeves
Roland Regoes

Barbara Rehermann
Jonathan Reichner
Alan Rein
William Reisen
Han Remaut
Bernhard Renard
Jyothi Rengarajan
Michelle Reniere
Rolf Renne
Martijn Rep
Jesus Requena
Arturo Reyes-Sandoval
Todd Reynolds
Kyu Rhee
Paula Ribeiro
Ruy Ribeiro
Andrew Rice
Stephen Rice
Kristy Richards
Thomas Richards
Christopher Richardson
Douglas Richman
Alan Rickinson
Michelle Riehle
Roland Riek
Arne Rietsch
Steven Riley
Guillermo Risatti
Amariliz Rivera
Fabian Rivera-Chavez
Nadia Roan
Michael Robek
Marjorie Robert-Guroff
Sally Roberts
Charles Robin
Christopher Robinson
Derrick Robinson
Harriet Robinson
Rosemary Rochford
Charles Rock
Daniel Rockey
Richard Roden
Isabel Roditi
Marcio Rodrigues
José F. Rodríguez
Mario Rodriguez-Perez
Andrew Roe
Paul Roepe
David Rogers
Stephen Rogerson
John Rohde
George Rohrmann
Richard Roller
Victor Romanowski
Pascale Romby
Julian Rood
Suzan Rooijakkers
Cliona Rooney
Ray Roos
Marilyn Roossinck
Michael Root
Patricia Rosa
Jason Rosch
Rebecca Rose
Ilan Rosenshine
Philip Rosenthal
Monica Roth
Stefan Rothenburg
Simon Rothenfusser
June Round
Christine Rouzioux
Aileen Rowan
David Rowlands
Polly Roy
Tania Rozario
Luis Rubio
Thomas Rudel
Gloria Rudenko
Rosa M Ruiz-Vázquez
Jürgen Ruland
Tillmann Rumenapf
Ruth Ruprecht
Colin Russell
David Russell
Brent Ryckman
Bernhard Ryffel
Agnieszka Rynda-Apple
Wang-Shick Ryu
Suraj Sable
Jonah Sacha
Ben Sadd
Jeroen Saeij
Asier Sáez-Cirión
Helen Saibil
Yusuke Saijo
Takeshi Saito
Maria Carla Saleh
Kevin Saliba
Maria Salvato
Karl Salzwedel
Amali Samarasinghe
Clare Sample

Jeffery Sample
John Samuelson
Thibault Sana
Veronica Sanchez
Rogier Sanders
Suzanne Sandmeyer
Rozanne Sandri-Goldin
Helene Sanfacon
Dominique Sanglard
Philip Santangelo
Mario Santiago
Erica Saphire
Martin Sapp
Saumendra Sarkar
Peter Sarnow
Peter Satir
Kei Sato
Yorifumi Satou
Quentin Sattentau
John-Demian Sauer
Ram Savan
Robert Sawers
Sara Sawyer
Charles Scanga
Marcel Schaaf
Timothy Schacker
Joanna Schaenman
William Schafer
Luis Schang
Hermann Schätzl
Troels Scheel
Jeffrey Schertzer
William Schief
Dieter Schifferli
John Schiller
Christian Schindler
Martin Schlee
Mark Schleiss
Larry Schlesinger
Patrick Schlievert
Dirk Schluter
Anthony Schmitt
Lutz Schmitt
Dirk Schnappinger
Petra Schneider
Tanja Schneider
Herman Scholthof
Anja Scholzen
Sebastian Schornack
Tony Schountz
Stacey Schultz-Cherry
Thomas Schulz
Erwin Schurr
Martin Schuster
Stephan Schwander
Ira Schwartz
Olivier Schwartz
Herbert Schweizer
Martin Schwemmle
Alan Scott
Phillip Scott
Rona Scott
Gavin Screaton
Thomas Scriba
Eileen Scully
Michael Seaman
Florent Sebbane
Peter Sebo
Robert Seder
Thomas Seebeck
Anna Seekatz
Rafick Sekaly
Bert Semler
Oliver Semmes
Susan Senogles
Irini Sereti
Ruth Serra-Moreno
Alessandro Sette
Stephanie Seveau
Wenwen Sha
William Shafer
Yechiel Shai
Libo Shan
Cynthia Sharma
Neelam Sharma-Walia
Paul Sharp
George Shaw
Lindsey Shaw
Megan Shaw
Samuel Shelburne III
Aimee Shen
Hao Shen
Qian-Hua Shen
Yuequan Shen
Donald Sheppard
Alan Sher
Nathan Sherer
Ethan Shevach
Meiqing Shi
Pei-Yong Shi
Chiaho Shih
Shin-Ru Shih

Ronald Shikiya
Sunny Shin
Joanna Shisler
Joseph Shlomai
Steven Short
Naglaa Shoukry
Hong-Bing Shu
Deepak Shukla
Howard Shuman
L. David Sibley
Tim Siegel
M. Sloan Siegrist
Paul Sigala
Philippe Silar
Lawrence Silbart
Robert Siliciano
Neal Silverman
Olivier Silvie
Jerry Simecka
Graham Simmons
Viviana Simon
Carmen Simón-Mateo
John Sinclair
Steven Singer
Upinder Singh
Amit Singhal
Photini Sinnis
Mark Siracusa
Rebecca Skalsky
Patrick Skelly
Shawn Skerrett
Jacek Skowronski
Claudio Slamovits
James Slauch
Barry Slobedman
Jason Slot
Geert Smant
James Smiley
Despina Smirlis
Davey Smith
David Smith
Gavin Smith
Geoffrey Smith
Greg Smith
Mitchell Smith
Terry Smith
Sigrun Smola
Ashleigh Smythe
Christopher Snyder
Magdalene So
Kenneth Söderhäll
Donald Sodora
Evgeni Sokurenko
Thierry Soldati
Jay Solnick
Holger Sondermann
Langzhou Song
Wenxia Song
Uwe Sonnewald
Kamoun Sophien
Joseph Sorg
Javier Sotillo
Graça Soveral
Jessica Soyer
Stijn Spaepen
Gerald Spaeth
Paul Spearman
Deborah Spector
Daniel Speiser
Juliet Spencer
Vanessa Sperandio
Christina Spiropoulou
Gary Splitter
Steven Spoel
Serena Spudich
Shiranee Sriskandan
Gary Stacey
Simona Stäger
Christina Stallings
Leonidas Stamatatos
Thomas Stamminger
Sarah Stanley
Danielle Stansic
Richard Stanton
Kenneth Stapleford
Michael Starnbach
John Steel
Chad Steele
Catherine Stein
Daniel Stein
William Steinbach
Jens Steinbrenner
Steffen Stenger
David Stephens
Robin Stephens
Noam Stern-Ginossar
Torsten Sterzenbach
Alasdair Steven
Brian Stevenson
Philip Stevenson
Adrie Steyn
Daniel Stieh

Brad Stiles
Monique Stins
Jose Stoute
Jonathan Stoye
Blair Strang
Klaus Strebel
Daniel Streblow
Natalie Strynadka
Frank Stubenrauch
Jason Stumhofer
Elena Stylianou
Lishan Su
Carlos Subauste
Malarkannan Subramaniam
Peter Sudbery
Brian Sullivan
Christopher Sullivan
Deborah Sullivan
William Sullivan
William Sullivan Jr.
Michael Summers
Jie Sun
Joseph Sun
Ren Sun
Yi Sun
George W. Sundin
Paula Sundstrom
Surachai Supattapone
Michael Surette
Witold Surewicz
Mehul Suthar
Susmit Suvas
Nobuhiro Suzuki
Staffan Svard
Joel Swanson
Luc Swevers
W. Edward Swords
Gulam Syed
David Symer
Moriah Szpara
Henry Tabel
Fabienne Tachini-Cottier
Frank Tacke
Frédéric Taieb
Nicholas Talbot
Yunhao Tan
Christoph Tang
Qiyi Tang
Xiaoyan Tang
Vera Tarakanova
Alexander Tarr
Ann Tate
Peter Tattersall
Norbert Tautz
John Tavis
Diane Taylor
Naomi Taylor
Terrie Taylor
John Teijaro
Antonio Teixeira
Luis Teixeira
Amalio Telenti
Alice Telesnitsky
Sam Telford III
Glenn Telling
Bhanu Telugu
Benjamin Tenoever
Mauricio Terebiznik
David Thanassi
Dimitris Thanos
Gaël Thébaud
Kevin Theis
Robert Thimme
Emmanuel Thomas
Gary Thomas
Matthew Thomas
Wellems Thomas
Richard Thompson
Hans Thordal-Christensen
David Thorley-Lawson
Adrian Thrasher
Scott Tibbetts
Leann Tilley
Jens Tilsner
Kyle Tipton
David Tobin
Rafael Toledo
Georgia Tomaras
Ana Tomás
Massimo Tommasino
Stephen Tompkins
Liang Tong
Shuping Tong
Christopher Tonkin
David Topham
Bruce Torbett
Jordi Torrelles
Alfredo Torres
Victor Torres
Domenico Tortorella
Zsolt Toth
Jean-Nicolas Tournier

Greg Towers
Paula Traktman
Erin Tran
Lydie Trautmann
Leonardo Travassos
Ana Traven
Steven Triezenberg
William Trimble
Lindsay Triplett
Emily Troemel
Billy Tsai
Anastasios Tsaousis
David Tscharke
Chien-Te Kent Tseng
Moriya Tsuji
Paul Tudzynski
Burkhard Tümmler
Bruce Turnbull
Joanne Turner
Keith Turner
Kenneth Tyler
Ioannis Tzanetakis
Massaro Ueti
Anne-Catrin Uhlemann
Christel Uittenbogaart
Buddy Ullman
Chris Upton
Constantin Urban
Stephan Urban
Edward Usherwood
Mart Ustav
Jude Uzonna
Monica Vaccari
Eeva Vainio
Ioannis Vakonakis
Barbara Valent
Susana Valente
Jesus Valenzuela
Bruce Vallance
Adrian Valli
Miguel Valvano
Frank van de Veerdonk
Guido Van den Ackerveken
Alex van der Bliek
Sjoerd van der Burg
Tom van der Poll
Adrianus van der Velden
Giel van Dooren
Thomas Van Dyke
Peter Van Esse
James Van Etten
Frank van Kuppeveld
Rene van Lier
Carine Van Lint
Mark van Raaij
Ronald Van Rij
Jos van Strijp
Russell Vance
Anne-Mieke Vandamme
Scott Vande Pol
Brian Vanderven
Russell Vangelder
Julien Varaldi
Steven Varga
Nikos Vasilakis
Subhash Vasudevan
Ashley Vaughan
Jefferson Vaughan
Jiri Vavra
Ronald Veazey
Jan-Willem Veening
Michele Vendruscolo
Harry Vennema
Patricia Veras
Jeanmarie Verchot
Nuria Verdaguer
Sergio Verjovski-Almeida
Subhash Verma
Rosanna Vescovini
Cecile Viboud
Toni Vidal-Puig
Leda Vieira
Dhanasekaran Vijaykrishna
Matam Vijay-Kumar
Boris Vinatzer
Joseph Vinetz
Sandhya Visweswariah
Eric Vivier
David Vocadlo
Christian Voigt
Veronika Von Messling
Martin Vordermeier
Rhonda Voskuhl
Martin Voskuil
Till Voss
Daniel Voth
Jatin Vyas
Andreas Wack
Stephen Waggoner
Ralf Wagner
Samuel Wagner
Sun Nyunt Wai

Aleksandra Walczak
Anna Wald
Matthew Waldor
Mark Walker
Thomas Walker
Daniel Wall
Jacco Wallinga
Pegine Walrad
Derek Walsh
Yisong Wan
Chengshu Wang
Guo-Liang Wang
Lin-Fa Wang
Ming-Bo Wang
Nian Wang
Taia Wang
Tian Wang
Xian-Bing Wang
Yan-Yi Wang
Z. Wang
Zonghua Wang
Tamding Wangdi
Andrew Ward
Honorine Ward
Matthew Wargo
Digby Warner
Steven Wasserman
Andrew Waters
Christopher Waters
Daniel Watterson
Joel Watts
Sing Sing Way
Pamela Wearsch
Richard Webby
Friedemann Weber
Heiner Wedemeyer
Christopher Weidenmaier
Jason Weinberg
Jeffrey Weiser
Jerrold Weiss
Louis Weiss
Susan Weiss
Winfried Weissenhorn
Matthew Weitzman
Matthew Welch
Sandra Weller
Melanie Wellington
Robert Welliver
Susanne Wells
Zhexing Wen
Wolfgang Weninger
Bregje Wertheim
Joel Wertheim
Catherine Werts
Susan Westmoreland
Robert Wheeler
Stephen Whisson
Denise Whitby
Judith White
K. Andrew White
A. White Jr.
Adrian Whitehouse
Jason Whitmire
James Whitney
Matthew Wiebe
Lüder Wiebusch
Philipp Wiemann
Emmanuel Wiertz
Clayton Wiley
Steven Wilhelm
Gavin Wilkinson
Gavin W.G. Wilkinson
Katalin Wilkinson
John Williams
Kenneth Williams
Matthew Williams
Mark Wills
Angus Wilson
Cara Wilson
Duncan Wilson
Heather Wilson
Joyce Wilson
Patrick Wilson
Richard Wilson
Sam Wilson
Van Wilson
Van G. Wilson
Jeffrey Wilusz
Eckard Wimmer
Wade Winkler
Sebastian Winter
Charles Wira
Alan Wolfe
Matthew Wolfgang
Hans Wolf-Watz
Thomas Wolpert
Adrian Wolstenholme
Christiane Wolz
Charles Wood
Thomas Wood
Floyd Wormley Jr.
Michael Worobey

Daniel Wozniak
Jens Wrammert
Gavin Wright
Terry Wright
Joseph Wu
Louisa Wu
Yuntao Wu
Zhijian Wu
Betty Wu-Hsieh
Felix Wussow
Marcel Wuthrich
Richard Wyatt
Chuanwu Xi
Zanxian Xia
Yan Xiang
Xiufang Xin
Zhou Xing
Zhiheng Xu
Chaoyang Xue
Timothy Yahr
Masahiro Yamashita
Nan Yan
Bing Yang
Priscilla Yang
Ruifu Yang
X. Frank Yang
Xi Yang
Xinzhen Yang
Zhi Yao
George Yap
Wendell Yarbrough
Robert Yarchoan
Felix Yarovinsky
Jun-Ichirou Yasunaga
Jian Ye
Sheng Ye
Michael Yeaman
Minkyung Yi
Fitnat Yildiz
Wayne Yokoyama
Timothy Yoshino
Jianxin You
Neil Young
Paul Young
Benjamin Youngblood
Jacob Yount
Xu Yu
Yan Yuan
Zheng-Hong Yuan
Jonathan Yuen
Kwok-Yung Yuen
Sung-Hwan Yun
Andrew Yurochko
Mark Zabel
Anna Zaidman-Rémy
Allan Zajac
Dirk Zajonc
Jianye Zang
Michael Zasloff
Fidel Zavala
Lauren Zenewicz
Bing Zhai
Kai Zhang
Bo Zhao
Jianhua Zhao
Jun Zhao
Kong-Nan Zhao
Yue Zhao
Jialin Zheng
Yong-Hui Zheng
Jin Zhong
Jiyong Zhou
Jun Zhou
Rui Zhou
Xueping Zhou
Fanxiu Zhu
Guan Zhu
Yongqun Zhu
Véronique Ziegler-Graff
Dan Zilberstein
Adam Zlotnick
Susan Zolla-Pazner
Lingyun Zou
Jeremy Zucker
Elina Zuniga
Li Zuo
Arturo Zychlinsky
